# Evolution and Heredity

**Published:** 1895-10

**Authors:** E. Symes Thompson

**Affiliations:** Gresham Professor


### Abstracts and Selections.

### Evolution and Heredity.

BY E. SYMES THOMPSON, M. D,
Gresham Professor.

The subject is a vast one. Heredity, or the inheritance of acquired characteristics, is one of the laws of Evolution. Heredity and Evolution are inseparable and inter dependent; they act together. Evolution without heredity would make every modification of form and character transient, whilst Heredity without Evolution would lead to never-changing monotony; the two forces (when combined) secure continuity and variation.

The doctrine of Evolution is the most sublime conception of our era. Darwin has been well named "the Newton of Natural

History." It is our privilege to have lived with Darwin. Sir Thomas Gresham, the noble founder of Gresham College, lived with Copernicus and Galileo, and realizing, as he did, the truth and the value of astronomical science, he founded the chair of astronomy now so ably filled by Professor Ledger. Galileo was compelled, by the authority of the church, to withdraw his assertion that the sun, and not the earth, was the center of our system, and many of us can remember the excitement and violent opposition that was raised when Darwin ventured to enunciate his great discovery, the doctrine of Evolution. Evolution is one of those grand generalizations that deserves to be placed by the side of gravitation. Isaac Newton showed not only that an apple falls to the ground by the force of gravitation, but that the same attractive force holds the sun, the moon and the planets in their course. This law was afterwards shown by Herschel to hold good for all the stars.

As the centrifugal force, due to the rapid rotation of the heavenly bodies, is controlled by the centripetal force of gravitation, so Evolution is kept under control by the restraining influence of the law of hereditary transmission.

Sir George Grove, also our contemporary, has discovered another great law of nature, the mutual correlation and convertibility of force, and has pointed out the great results that follow from the controlling action of opposing forces.

Before the discovery of these great principles the sciences were in their infancy. The seeker after truth was met everywhere by problems which he could not explain or understand. The discovery of these truths has made easy what was before enigmatical, and has profoundly increased our interest in nature, and enabled us to appreciate in a measure the mode of operation of God in nature.

The telescope, which has brought within our reach myriads of worlds whose very existence we sould not otherwise surmise, has also enabled us to fathom the nature and the causes of their movements, and though we can not see the medium through which gravitation acts, we are able to reason about it, and to account for the movements of the heavenly bodies.

The spectroscope again has enabled us, as it were, to put them into the crucible, and to define the composition of the invisible stars. We are thus enabled to come within touch of radiant suns whose distance is well nigh immeasurable, and to discern that far greater influence is exerted on our atmosphere by the stars that are invisible to the unaided eye than by those we can see. Of the light, heat and chemical force that emanate from our sun, but an infinitesimal part find its way to the planets and comets that form its solar system, 2,200,000,000 times as much force is (so far as we know) wasted in space. Can we believe that it is really "wasted"? Is it not more consonant with our ideas of creative wisdom and versatility to assume with Lord Kelvin that these "wasted rays" are utilized in causing the formation of "vortex rings" in the circumambient ether, which rings formed by motion compose the atoms out of which all material bodies are built up. Enlightened chemistry tells us of a grouping of atoms in accordance with certain fixed laws, and the surmise has been ventured upon that hydrogen, the lightest of the gases, is really a metal, and, more than this, that it is a fundamental metal, from the elaboration of which all the others are an outcome.

From the study of the heavenly bodies we learn that the law of Evolution extends to them, for we see them in various stages of growth and development.

First the nebula, not yet aggregated into definite form; next the globe in a state of gaseous ignition; then gradual consolidation, white heat passing into red heat, and, as the body is cooled down, it attains first the condition in which our sun is now, and then that of our earth.

Here geology comes in to confirm the same tale, and we learn that the igneous rocks became gradually cooler, the boiling rain left its indented impress on the mud since hardened, and now used as flag stones in our streets, the metamorphic rocks followed by the sedimentary, in primary, secondary and tertiary times, ushered in our own era.

Thus we are enabled to sketch the life-history of the universe, the solar system and the earth, and are prepared to find in the stratified rocks the story of Evolution written for us in characters at first as enigmatic as Egyptian hieroglyphics, but now, read by the light, as it were, of the Rosette Stone, and made so clear that he that runs may read, the demonstration is so full as to command assent, although many details are left to be filled in by new discovery.

Palæontology is not the only science which throws light on Darwin's great generalization. Archæology is ever bringing new evidence before us. The study of the forms of the skulls and bones of different nations tells the same tale, and when we come from primitive man in the stone age, the iron age and the bronze age to the historical era, the story is ever the same. In Egypt, we are taken back for sixty centuries, and are brought into intimate relation, by means of the sermons preached to us in hieroglyphic characters on the temple walls, with the great men of the past, whilst the bones and mummified remains of Rameses the Great and the kings of earlier dynasties, thousands of years before the time of the Exodus, all confirm the truth of the great underlying principle, and show us clearly that among the laws by which the Creator delights to act are the law of Evolution, the law of gravitation, and the law of correlation, all of which are alike ubiquitous.

We see everywhere unity of structure. The universe is an organism bound by the cords of gravitation, developing by the law of Evolution, and having every part intimately correlated with every other, action and reaction, and the mutual influences of opposing forces are everywhere to be traced. Whilst the telescope shows us these forces in the universe, the microscope reveals them in minute organisms, as much beyond the reach of the unaided eye as are distant nebulæ. We are too utterly mundane in our thoughts to be able easily to realize that the radiant suns that fill the universe are like the cells of a plant or animal. The plant may be of microscopic size, and the distances between its composing cells almost inappreciable, while the distances that separate the suns of the universe may be such as to require centuries for a ray of light to pass between them, yet they are held together by the forces of gravitation, evolution and chemical action. Some of the stars formed in the dim ages of the past are still young and of primitive development, so too, in the animal and vegetable world we meet with many forms which have existed since the first stratified rocks were deposited, and are still simple in structure and unchanged in character. Such are the earthworm, the coral, the fungi, and the lichens; they remain almost unchanged, and fulfill their function, which could not be better served by higher and more elaborated organisms.

Meanwhile, as the suns that were once little more than whorls of hydrogen, have consolidated, are found, by the spectrum, to be composed of metal of every kind, and, cooling, become the home of higher organized beings; so may we trace from the lowly forms an ever increasing elaboration adapted to the more com plex environments.

These organisms are generally found to be more diverse the more widely they are separated. Yet this is not always so. New Zealand and Australia are but a few hundred miles apsrt, yet the fauna and flora of each are utterly unlike. Madagascar has but a narrow channel between it and the African coast, yet the living forms are most diverse. An enormous interval divides England from Japan, yet the creatures inhabiting the two are more nearly allied than are those of New Zealand and Australia, Madagascar and Africa. The reason is found to be this, that a deep ocean separates these near countries, whereas no deep ocean intervenes between England and Japan. In the early Tertiary period, no Straits of Dover divided the British Islands from the continent of Europe, and no Mediterranean Sea prevented animals from Africa from walking across to England, to Iceland, or even to Greenland.

The Mollusca of the Philippine Islands, as recently shown by Mr. Cooke, in his charming work, teach the same lesson. Those islands which have but a shallow sea between them have the same creatures, but those which are surrounded by deep water have flowers and animals of their own.

Many problems are suggested when we test the distribution of animals which, when the answer is discovered, are found to be no longer problems, but illustrations of known laws. Take, for instance, the tapirs of Borneo and Tropical America. How did they travel from such distant lands and leave no representatives in intervening places? The answer is this: Tapirs were once widely distributed, but, in the struggle for existence, they were thrust out by more powerful mammals, and now hold on an existence only in those unwholesome tropical swamps where no more sensible or cultivated animal would care to sojourn.

The Marsupials were not always confined to Australia, they were once widespread and almost universal, but the mammalia beat them out of the field and they only lived on in an island where no mammalia were to be found. The oceanic islands in which the mammalia were absent have shown a strange tendency to develop large wingless birds, some of these, like the Moa and the Dodo, are now extinct. It is curious that the New Zealand birds, though large, are cowardly, and an English thrush or blackbird will frighten away birds much larger than itself.

When Darwin first published his observations and pointed out that the changes he had proved to exist in the varieties of pigeons, violets, etc., were not limited to varieties but extended also to species, and showed that the principle held good for the whole race, many persons were ready to accept the truth of the view to a limited but not to a large extent. Before the time of Darwin and of Wallace, it was supposed that every kind of animal and of plant was specially created, but observation and experiment led to the irresistible conclusion that all the varying forms were due to a series of slow developments taking place under the law of natural selection. Perhaps no more striking proof of the universality of this law has been adduced than is to be drawn from the Ornithorhyncus Paradoxus, or from the so-called Ant-eater of New Guinea. This strange creature has the characteristics of the bird, the reptile, and the mammal. It maintains to this day a character implanted on its ancestors at a period of the world's history when the distinctions between the great classes of reptiles, birds, and mammals did not exist. Strange, indeed, it is to find a creature that lays eggs and suckles its young. It has the shoulder-girdle and heart of a reptile, and, most strange of all, it has teeth, which indeed are not used, being shed in very early life, identical with those of the Labrynthodon, an animal that has been extinct since the secondary Geologic period. The existence of such creatures as these goes far to prove that Darwinism does not merely account for varieties and genera, but that it actually extends to the great sub-divisions of animal creation.

We can watch the formation of varieties by artificial selection and sometimes by natural selection, as in the case lately desribed of the Tits of the Canary Islands. A new variety of Tit was first discovered on one island of the group, and on a second island of the same group a sub-variety was found, and on a third island another sub-variety. A pair of Tits long ago were carried to one of these islands, the descendants bred "in and in" until all the salient characteristics of the original pair, uncontrolled by intermixture with other families, became so emphasized in the descendants that eventually a distinct variety was formed. After a time, owing perhaps to an exceptional condition of wind, one pair of these birds were blown on to each of the other islands, which were too distant for ordinary inter-communication; and thus the characteristics of each young couple begun again to be pronounced, so that two sub-varieties were established, manifestly related to, although distinct from, the parental stock.

Such varieties may be best followed on oceanic islands far away from other land, and throw a flood of light on the controlling power of hereditary influence in arresting the otherwise great variations wrought in time by the unchecked action of the law of Evolution.

The history of the development of the horse, as worked out by Professor Marsh of Yale College, and expounded by Professor Huxley, illustrates well the progressive evolution of this useful animal. Prof. March found thirty species of fossil equidae. The oldest representative of the horse is the diminutive Eohippus of the Lower Eocene beds, which in the structure of its feet and teeth unmistakably indicates that the direct ancestral line of the modern horse has already separated from the other odd-toed ungulates. The Orchippus of the Higher Eocene intervenes between this and the Mesohippus of the early Miocene deposits. This, instead of being like the Eohippus, the size of a dog, is as large as a sheep, the four toes are reduced to three. The Miohippus of the Upper Miocene leads directly to the Protohippus of the Lower Pliocene, which is more equine and as large as an ass. Of the three toes only the middle one, which corresponds with the single toe of the horse, reaches the ground.

The Pliohippus of the Pliocene is the last stage; it has lost the small lateral hooflets and is very equine. But it is only in the Upper Pliocene that the true Aquus appears; it roamed over the whole of North and South America in the post-tertiary times and, strange to say, it soon became extinct.

Well may Huxley say that this demonstration of Evolution rests on a secure a foundation as did the Copernican theory of the motions or the heavenly bodies at the time of its promulgation.

The development of the true deer gives an equally unmistakable proof of evolution.

The arguments formerly adduced against Evolution that geology supplied no evidence of the gradual development of organic forms, has been swept away with fuller knowledge. Sudden appearances suggesting new creations are deceptive, and arise from the fact that the conditions favorable to the fossilization of animals occur but rarely, and only in limited areas.

In the case of man we have proofs of his existence during and prior to the Glacial epoch, but we have no evidence as to his association with the anthropoid apes; indeed, when we seek for the closest analogue as regards bones, brain, etc., we find it, not in the chimpanzee or the gorilla, but in the lemuridæ of an early geologic epoch. It is not that we have lost the "missing link," but that a great many links are lacking to complete the chain.

Wallace thinks it impossible but that ample remains of Miocene and Pliocene men do exist buried in the earth's crust, to be one day discovered. Just as men escaped from Pompeii during the destruction of the city, so doubtless men, by reason of their intelligence, have been able to escape from engulfing mud or scoria which has preserved for us the relics of lower animals.

The world has been but little explored in that portion of it where the cradle of our race may be supposed to exist. The story of genus homo is not yet written as is that of the equidæ above detailed. Evidence is entirely lacking as to intermediate forms. Even if we should eventually complete the chain, so far as anatomical structure is concerned, we can not expect that geology will supply us with evidence as to the time and nature of the intellectual development which distinguishes our race, still less of the powers and the potentialities which are in store for it. Our sensuous or scientific perceptions can give us no clue to the mystery of vital processes. To the senses of touch, sight, hearing these are alike inaccessible, but though hidden from the senses, they are not unimaginable by the intellect. We know something of natural bodies and of physical forces, but we know something, too, of the immaterial energy which dominates and directs them. We are at the same time both material and immaterial; and when we regard the various forms, animal and vegetable, we can hardly deny, even to the lowest, the possession of an immaterial directive principle which renders intelligible, though it does not explain, heredity, and the other processes of life. The law of Evolution plainly provided for a succession of transitory existences, but makes no further or more enduring provision.

The sketchy survey we have given of the inheritance of acquired characteristics and the relation Evolution bears to the other laws of nature does not seem to suggest that an attitude of confident self-complacence is suited for the man of science. The laws of Evolution and of gravitation were in action æons before Copernicus or Darwin or Newton. The worlds were evolving, before the telescope enabled us to see them, the spectroscope to test their composition, and before we had learned to prepare a film upon which invisible stars could photograph themselves. The lowest forms of animal and vegetable life were struggling upwards to higher manifestations, the lycopods of the coal measures had worked onwards till the oak and the beech took their place. The fish had given place to the reptile, the bird to the mammal, before the Book of Geology was opened. The chalk was formed of the skeletons of millions of living creatures before a microscope was made to show their existence, and it is but lately that this instrument has brought to light the minute organisms which we now know are responsible for many diseases. Even here the microscopist can not rest content, for, without the chemist, he would know nothing of the Ptomaines, which, as it were by the action of a ferment, are evolved by the microbe.

If this is true of material forces, it is equally true of the immaterial. When we consider the diversity of conclusions on all subjects which men of ability form, it is obvious that in any particular human mind there is no criterion by which truth is to be measured, caution and modesty are alike needed, and a man must, indeed, be vain-glorious if he regards his own mind as the ultimate standard of all truth. The greatest men, occasionally, make the greatest mistakes, because they deal with the greatest subjects. When we concentrate our attention on one law of nature, we must not worship it. The traveler in the dense forest of Brazil, seeing the palm tree which had turned itself into a creeper, and, growing up a tree trunk, had forced itself through the entangling foliage and then dropped its ærial root 130 feet to the ground, declared that Evolution could not have accomplished this, but that the plant possessed a true "instinct." When Darwin contemplated the evolution of the eye from its primitive form, the very thought "gave him a cold shiver." These and such like difficulties need not trouble us if, instead of regarding "Law" as a cold abstraction, outside the realm of supervision or modification, we look upon it as the method by which creative wisdom continually adjusts to their environment the forces which act on material things. The materialist can see nothing but matter, the metaphysician nothing but mind, whilst the man of spiritual insight is tempted to despise every gift but his own. Let it be our aim. as humble seekers after truth, to utilize to the utmost every gift, and to remember that those who consecrate their lives to one line of enquiry are doing their best for the advancement of truth, though they may be only trustworthy guides in that part of the great field of knowledge which is within their ken.

From things finite we can not judge of the eternal purposes of the Creator. God, in nature, is shown to exercise a power of amazing fertility, transcending time and space, but finite as regards the worlds and all that live upon them.—*Humanitarian.*

